# Reduction of procedure times in routine clinical practice with Compressed SENSE magnetic resonance imaging technique

**DOI:** 10.1371/journal.pone.0214887

**Published:** 2019-04-12

**Authors:** Elisabeth Sartoretti, Thomas Sartoretti, Christoph Binkert, Arash Najafi, Árpád Schwenk, Martin Hinnen, Luuk van Smoorenburg, Barbara Eichenberger, Sabine Sartoretti-Schefer

**Affiliations:** 1 Institute of Radiology and Nuclear Medicine, Kantonsspital Winterthur, Winterthur, Switzerland; 2 Faculty of Medicine, University of Zurich, Zurich, Switzerland; Medical University of Vienna, AUSTRIA

## Abstract

**Objectives:**

Acceleration of MR sequences beyond current parallel imaging techniques is possible with the Compressed SENSE technique that has recently become available for 1.5 and 3 Tesla scanners, for nearly all image contrasts and for 2D and 3D sequences. The impact of this technique on examination timing parameters and MR protocols in a clinical setting was investigated in this retrospective study.

**Material and methods:**

A numerical analysis of the examination timing parameters (scan time, exam time, procedure time, interscan delay time, changeover time, nonscan time) based on the MR protocols of 6 different body regions (brain, knee, lumbar spine, breast, shoulder) using MR log files was performed and the total number of examinations acquired from January to April both in 2017 and 2018 on a 1.5 T MR scanner was registered. Percentages, box plots and unpaired two-sided t tests were obtained for statistical evaluation.

**Results:**

All examination timing parameters of the six anatomical regions analysed were significantly shortened after implementation of Compressed SENSE. On average, scan times were accelerated by 20.2% (p<0.0001) while procedure times were shortened by 16% (p<0.0001). Considering all anatomical regions and all MR protocols, 27% more examinations were performed over the same 4 month period in 2018 compared to 2017.

**Conclusion:**

Compressed SENSE allows for a significant acceleration of MR examinations and a considerable increase in the total number of MR examinations is possible.

## Introduction

MR imaging is an established yet time-consuming radiological imaging technique. Thus due to high demand, limited patient comfort, economic pressure and the quest to improve image resolution without prolonging image acquisition, efforts have been made to shorten image acquisition times.

Recently, compressed sensing technologies that serve this purpose have become commercially available [1–9]. In this publication, Compressed SENSE was applied [2]. Compressed SENSE is a combination of the parallel imaging technique SENSE (sensitivity encoding) together with compressed sensing [3–9]. In SENSE-based acceleration, uniform undersampling strategies in the k-space domain are applied to reduce the amount of data sampled [3]. This allows for a reduction of data acquisition times. Information from the different elements in the receive coil is used to create an image from the subsampled data. The total SENSE acceleration factor is limited by the coil geometry. Compressed sensing aims to overcome this limitation by allowing incoherent subsampling schemes, acquiring less data than in SENSE undersampling schemes. This results in structured noise patterns that can be removed by transforming these images to a domain in which they may be sparsely represented, such as a wavelet domain. In this domain sparse information from the relevant structures in the images can easily be separated from the noise (so called denoising). In the Compressed SENSE implementation a variable density subsampling scheme was combined with a reconstruction algorithm that allows the combination of wavelet transformation of compressed sensing with coil information of SENSE [2].

Compressed SENSE is field strength independent; it may be applied to nearly all image contrasts with Cartesian acquisition schemes in all anatomies. Two parameters have to be optimized for each sequence individually; the Compressed SENSE reduction factor determines the acceleration of the sequence and thus the scan duration. The denoising level influences the amount of denoising in the wavelet domain and therewith changes the appearance of the images. A weak denoising level leads to a noisier image impression than a medium or strong denoising level, while stronger denoising produces a smoother image appearance [2].

Compressed SENSE is supposed to be capable of a reduction of 2D and 3D acquisition times by up to 50% and a reduction of 45% of the exam time in complete brain and cardiac examinations and of 25% in spine and musculoskeletal examinations in a tailored setting compared to identical acquisitions without Compressed SENSE technique but with standard SENSE-accelerated exams [2]. In this study, we aimed to investigate if it is possible to reach the same amounts of time reduction in clinical practice. More importantly, we investigated whether this results in the possibility of reducing overall procedure times.

In a clinical setting not only the scan duration but also the spatial resolution of the images acquired is important. Small imaging details that are often clues to the underlying diagnosis are often only detected on high-resolution images. Therefore, the use of Compressed SENSE is not limited to an acceleration of routine MR sequences but it is also used to increase the spatial resolution of sequences without increasing the scan time. Thus, the technique allows to redesign imaging protocols and to adjust workflow processes without any compromise in image quality and diagnostic capability of the images acquired.

Our department has used the technique since autumn 2017. We investigated the impact of Compressed SENSE on the time reduction of examination timing parameters and correspondingly on the shortening of our MR protocols and procedure times as well as on the amount of daily MR examinations performed over a period of 4 months in a large clinical radiological setting with 25 board certified radiologists. We performed this analysis after careful re-optimization of the standard imaging protocols for six anatomical areas (brain, knee, lumbar spine, wrist, breast and shoulder). In some protocols Compressed SENSE was purely used for acceleration, while in other protocols 3D acquisitions replaced 2D scans acquired in multiple orientations. The MR modality log files were used to assess the impact of Compressed SENSE on our radiological MRI workflow.

## Materials and methods

For this retrospective study no ethical approval from the institutional review board (Cantonal Ethical Committee Zürich) was required because firstly, protocol optimization leading to reduction of procedure times in MR examinations is performed as part of regular quality control and thus does not require any ethical approval. As for the figures Informed written consent was obtained by the three patients, as specified by the institutional review board. All data used in this study was fully anonymized before it was accessed and analysed.

This study consisted of two parts; first, the MR imaging protocols were optimized after the introduction of Compressed SENSE. Subsequently, the log files of the system were analysed to investigate the effect on several relevant timing parameters. All exams in this study were performed on an Ingenia 1.5 T MR scanner (Philips, Best, the Netherlands).

### Protocol adjustments and optimization

Prior to the log file analysis period, clinical protocols were adjusted to incorporate Compressed SENSE. Each neurological (brain), musculoskeletal (knee, lumbar spine, wrist and shoulder) and breast MR protocol in our department was reevaluated to include, exclude or modify sequences based on the new scan durations of the sequences, with the aim either to shorten the exam time or to improve the image resolution without prolonging the exam time. Individual protocols were either shortened in exam time as was done in standard brain, breast and shoulder protocol or Compressed SENSE was used to increase spatial resolution in the newly introduced 3D sequences as was done in the knee, wrist and lumbar spine exams. This was always performed in such a way that the scan time was equal or less than the original SENSE-accelerated sequence.

### Protocol optimizations for shorter exam times in brain, breast and shoulder imaging

Our standard brain MR protocol consists of precontrast 2D T1 FFE, DWI, 2D MultiVane TSE T2, 3D SWI, 3D FLAIR and postcontrast 3D T1 mDIXON TFE. Wherever possible, Compressed SENSE was applied to reduce acquisition times. Furthermore, Compressed SENSE was used to accelerate brain sequences that are not used in this standard brain protocol, such as 3D T1 TFE, 3D DIR, 3D T1 black blood TSE, 3D TOF MR-angiography and 3D PC venography.

For all sequences, the reduction factor (named Compressed SENSE factor) and the denoising level were carefully chosen by three (neuro)radiologists and resulted in an identical image impression as in scans obtained without Compressed SENSE.

A similar strategy was applied in breast and shoulder imaging.

All sequences were scanned in volunteers. Each sequence was scanned several times starting with a baseline scan without applying Compressed SENSE followed by several scans with steadily increasing Compressed SENSE factor. The Compressed SENSE factor was always increased by approximately 1 unit up to a maximum of a factor of 10. The images were then independently assessed by three board certified (neuro)radiologists who compared the image impression to the baseline scan. The highest possible Compressed SENSE factor resulting in identical image impression compared to the baseline scan was then chosen in agreement.

After having established the Compressed SENSE factor, the same method was applied to determine the denosing level by scanning the sequence with the Compressed SENSE factor while varying the denoising level.

To further validate the chosen Compressed SENSE factors and denoising levels every sequence was scanned with and without Compressed SENSE in ten patients. If image impression was then not deemed comparable, volunteer scans were repeated and a lower Compressed SENSE factor (possibly with a different denoising level) was chosen. This procedure was repeated several times until the images of every sequence were rated indistinguishable from the original baseline sequence.

### Protocol optimization for acceleration and improved resolution in lumbar spine, knee and wrist exams

In the original protocols for lumbar spine, knee and wrist similar 2D scans were acquired in multiple orientations. In the new protocols, these scans were replaced by a single 3D scan with a similar image contrast and isotropic or near-isotropic resolution, allowing multiplanar reconstructions in different orientations. The 3D sequences were secondarily accelerated with Compressed SENSE.

The same approach was applied in several other protocols (cervical spine, brachial and sacral plexus, intracranial vessel wall, inner ear and hypophysis), of which the performance was not further investigated in this study in detail.

### Log file analysis

**Fig 1:**
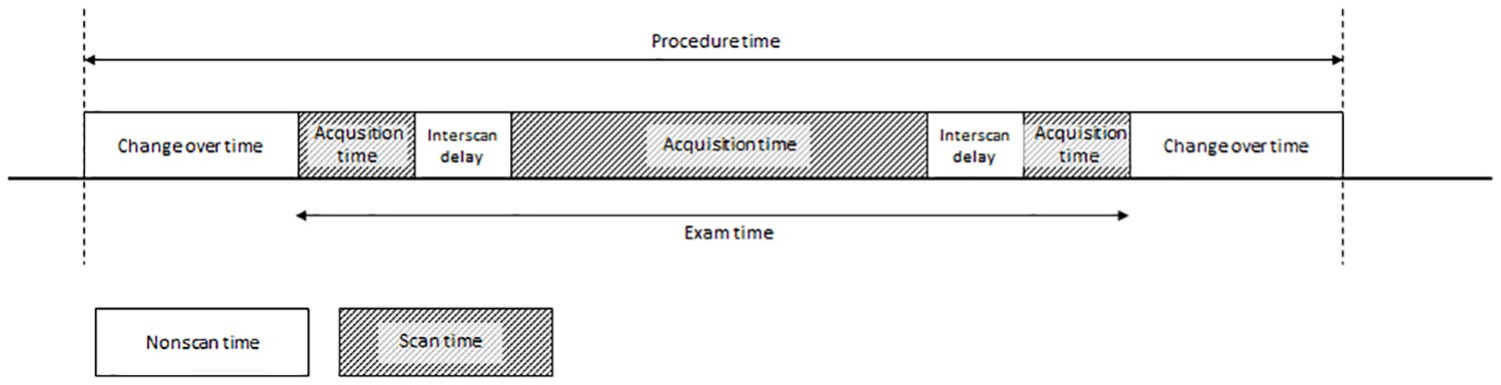
Graphical representation of examination timing parameters in a single patient. Procedure time is the duration the patient is in the scanner room, exam time is the sum of actual scan time (data acquisition) and inter scan delays such as planning of imaging stacks, communication with the patient and admission of contrast agent. The changeover time is the time required to put the patient on and off the table. Scan time is equivalent to the periods that the scanner is acquiring data (dashed blocks), while non-scan time is defined as all period where the scanner is not acquiring data (blank blocks).

All MR examinations (obtained without Compressed SENSE) from 1^st^ January 2017 to 30^th^ April in 2017 as well as in in the same period in 2018 (obtained with Compressed SENSE) were analysed. The MR modality log files were used for analysis of examination timing parameters (see below and Fig 1 for definitions). For each anatomical region several different MR protocols exist that try to answer various clinical problems, however usually the standard protocol is the most frequently applied protocol in each body region.

An evaluation of the following data was performed:

1. The MR examination mix defined as the number of MR examinations related to different body parts was extracted from the log files.
2. From the scanner protocols (exam cards) the following parameters were derived: the scan time of the six standard MR protocols acquired in six anatomical regions (brain, knee, lumbar spine, breast, wrist and shoulder), obtained without and with Compressed SENSE. The percentage of the scan time acceleration by Compressed SENSE and the corresponding sequence parameters of these individual sequences.
3. The average examination timing parameters for MR protocols of the six anatomical regions (brain, knee, lumbar spine, breast, wrist and shoulder) were derived from log files.
4. The average examination timing parameters for all MR examinations in the selected time periods were derived from log files.
5. The total number of MR examinations obtained in the selected time periods was derived from log files.
6. The impact of the Compressed SENSE technique on the daily radiological workflow was analysed based on the data obtained above.

Examination timing parameters: Any MR protocol consists of several sequences that are scanned consecutively. The time that is necessary to scan an individual sequence is called acquisition time. Further necessary scans such as localizing scans and ref scans are part of the MR protocol. The scan time is the sum of all the acquisition times of the different sequences of a specific MR protocol. The interscan delay time defines the time between two sequences, which amongst others can relate to planning of the location of image acquisition, interaction with the patient and admission of a contrast agent. The total exam time consists of the scan time and the interscan delay time. The changeover time is the time between the end of an MR examination and the start of the following examination. The sum of the exam time and changeover time is defined as the total procedure time. The nonscan time is defined as the sum of the interscan delay time and the changeover time (Fig 1).

MRI examinations were performed between 07.30 am to 8 pm from Monday to Friday (emergency exams on the weekends were not taken into account).

### Statistics

For data analysis percentages and box plots were used. The boxplots visualize five summary statistics, namely the median, two hinges and two whiskers and all "outlying" points individually (thus points beyond the end of the whiskers). The lower and upper hinges correspond to the first and third quartiles (the 25th and 75th percentiles). The whiskers extend from the hinge to the largest value no further than 1.5 * IQR from the hinge (where IQR is the interquartile range, or distance between the first and third quartiles).

Furthermore, to assess differences of certain time intervals, unpaired two-sided t-tests on a 5% significance level were used. Within figures significant p-values were underlined.

## Results

### Protocol optimization

**Fig 2:**
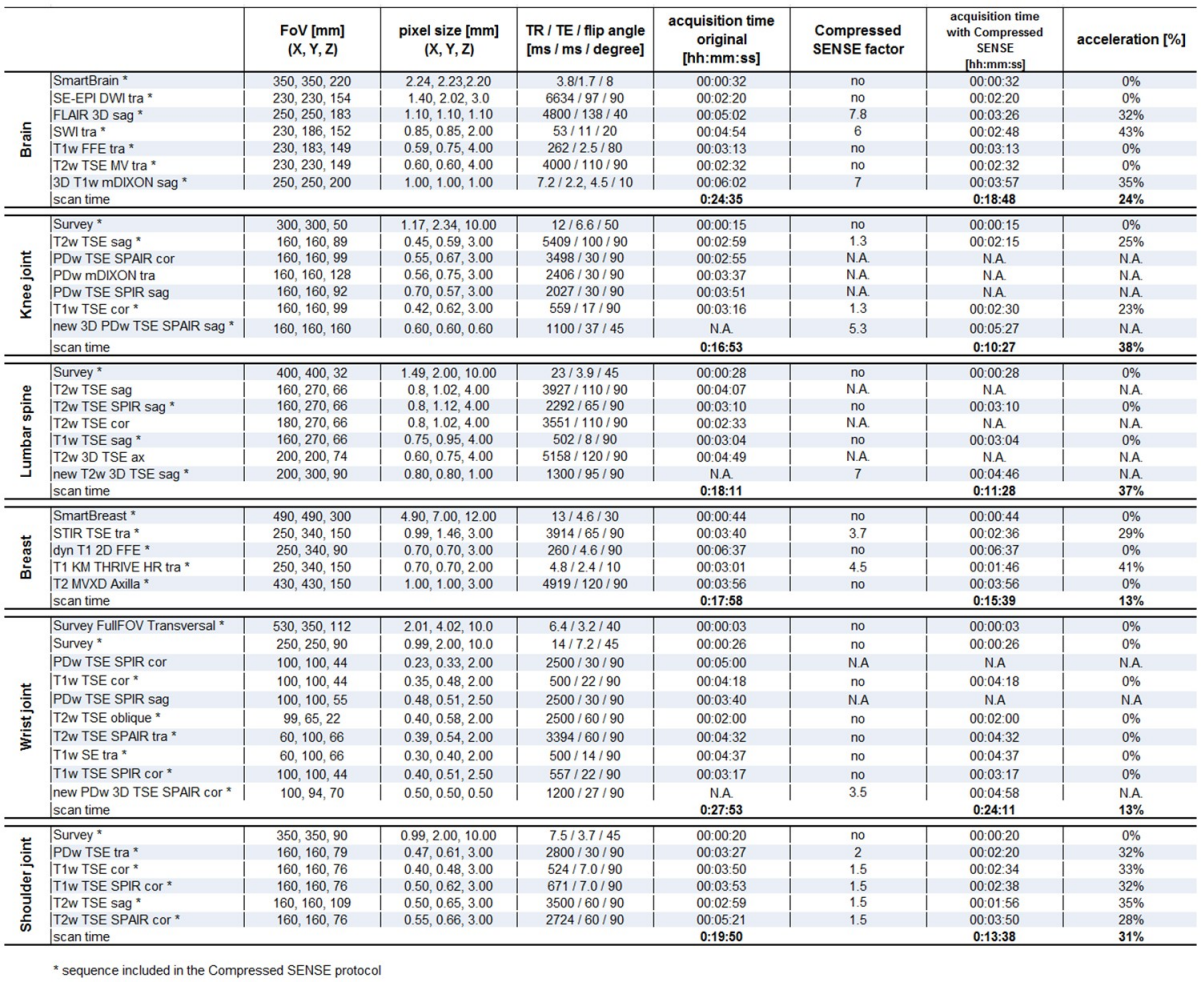
MR scanning parameters, times of individual sequences and the total exam prior to and after introduction of Compressed SENSE. FOV, resolution and most important scan parameters (TE / TR / flip angle) of the exams and the timings of the individual sequences and the total exam as performed prior to and after the introduction of Compressed SENSE are mentioned. The Compressed SENSE reduction factors are described where applicable. The total acceleration factor of a single sequence as well as of the full exam are given. Note that for brain, breast and shoulder Compressed SENSE was used for acceleration only, while in case of knee, lumbar spine and wrist a hybrid approach was chosen; in some sequences Compressed SENSE was used for acceleration, while other 2D scans were replaced by a (near-)isotropic 3D sequence with a similar weighting. “N.A.” means “not available” and is used if this sequence was not acquired after optimization of MR protocol with the Compressed SENSE technique. “No” means, that this sequence was acquired without Compressed SENSE technique in the original and the modified MR protocol.

Protocol optimization resulted in updated exam protocols, of which critical imaging parameters as well as scan times are depicted in Fig 2.

**Fig 3:**
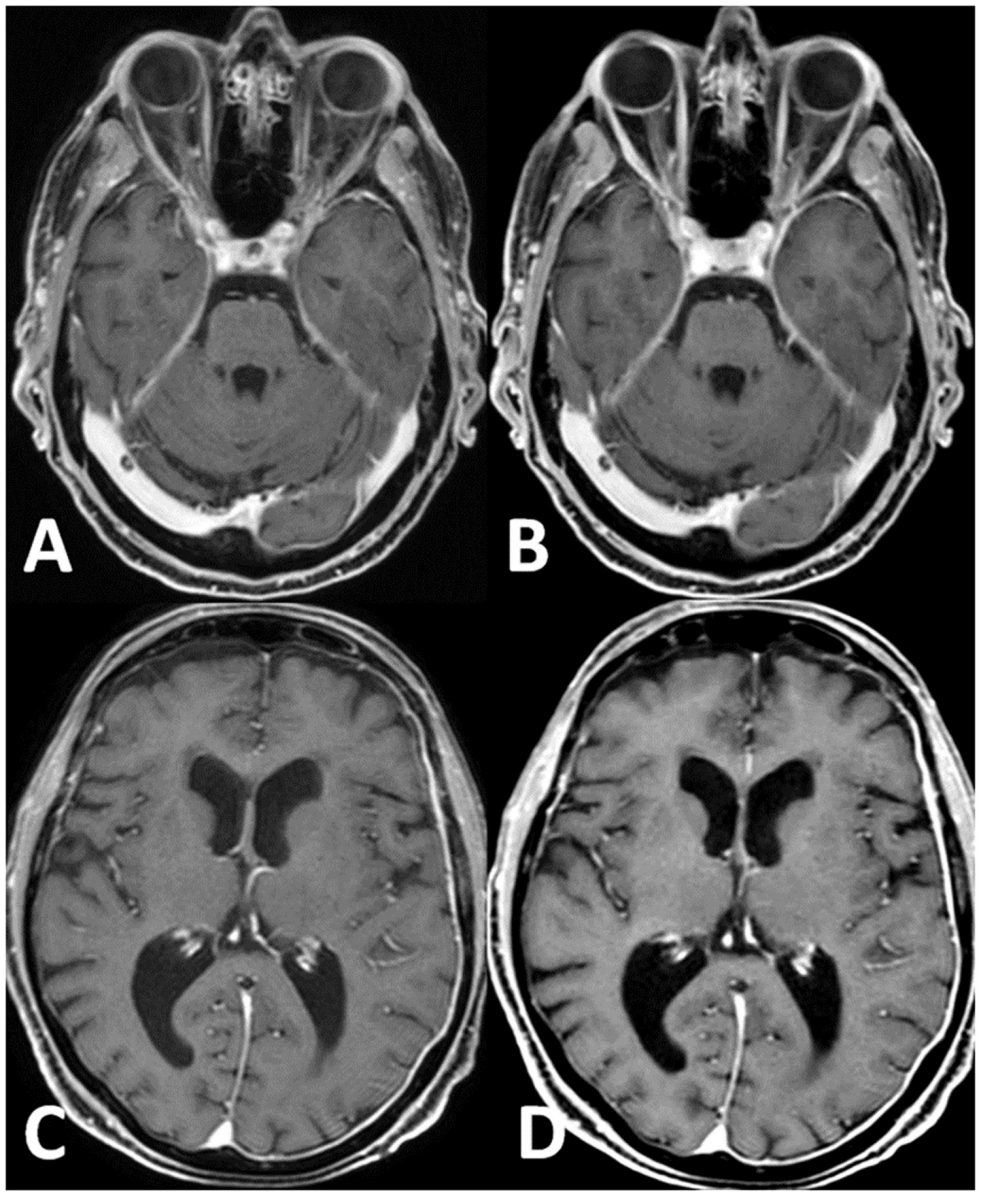
Illustration of image contrast achieved without and with Compressed SENSE in brain on 3D T1 m-Dixon TFE post-contrast images. Transverse image reconstructions of a sagittally scanned 3D T1 m-Dixon TFE postcontrast sequence acquired without (A and C) and with Compressed SENSE (B and D). A Compressed SENSE reduction factor of 7 was chosen. The image impression is comparable with and without Compressed SENSE.

**Fig 4:**
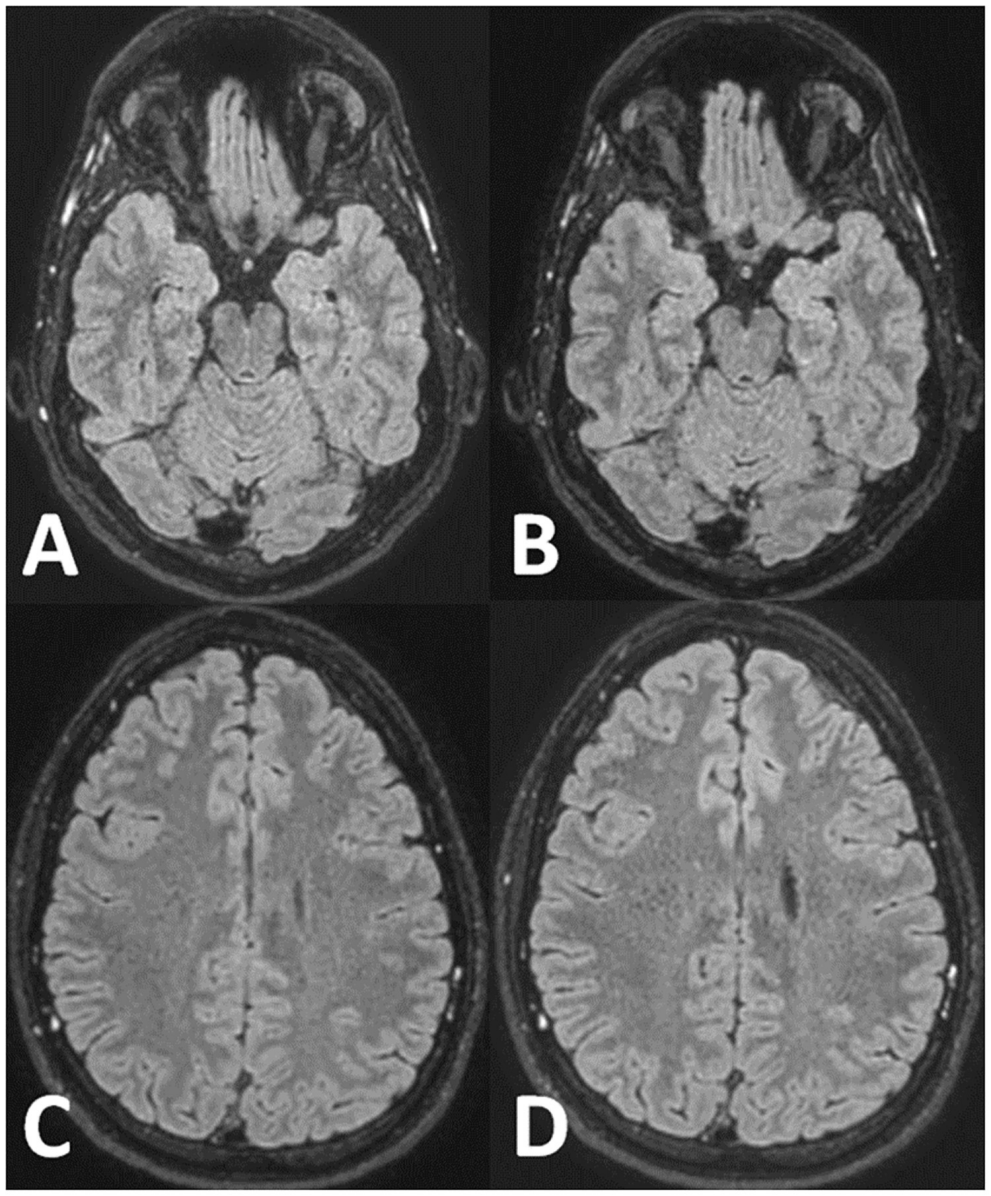
Illustration of image contrast achieved without and with Compressed SENSE in brain on 3D FLAIR images. Transverse image reconstructions of a sagittally scanned 3D FLAIR sequence acquired without (A and C) and with Compressed SENSE (B and D). A Compressed SENSE reduction factor of 7.8 was chosen. Comparable image impression with and without Compressed SENSE.

In brain, breast and shoulder joint Compressed SENSE was used for pure acceleration of the sequences wherever this was applicable and appropriate. In Figs 3 and 4 MR images prior to and after implementation are displayed to illustrate the similarity in image appearance of the original and the accelerated protocols.

**Fig 5:**
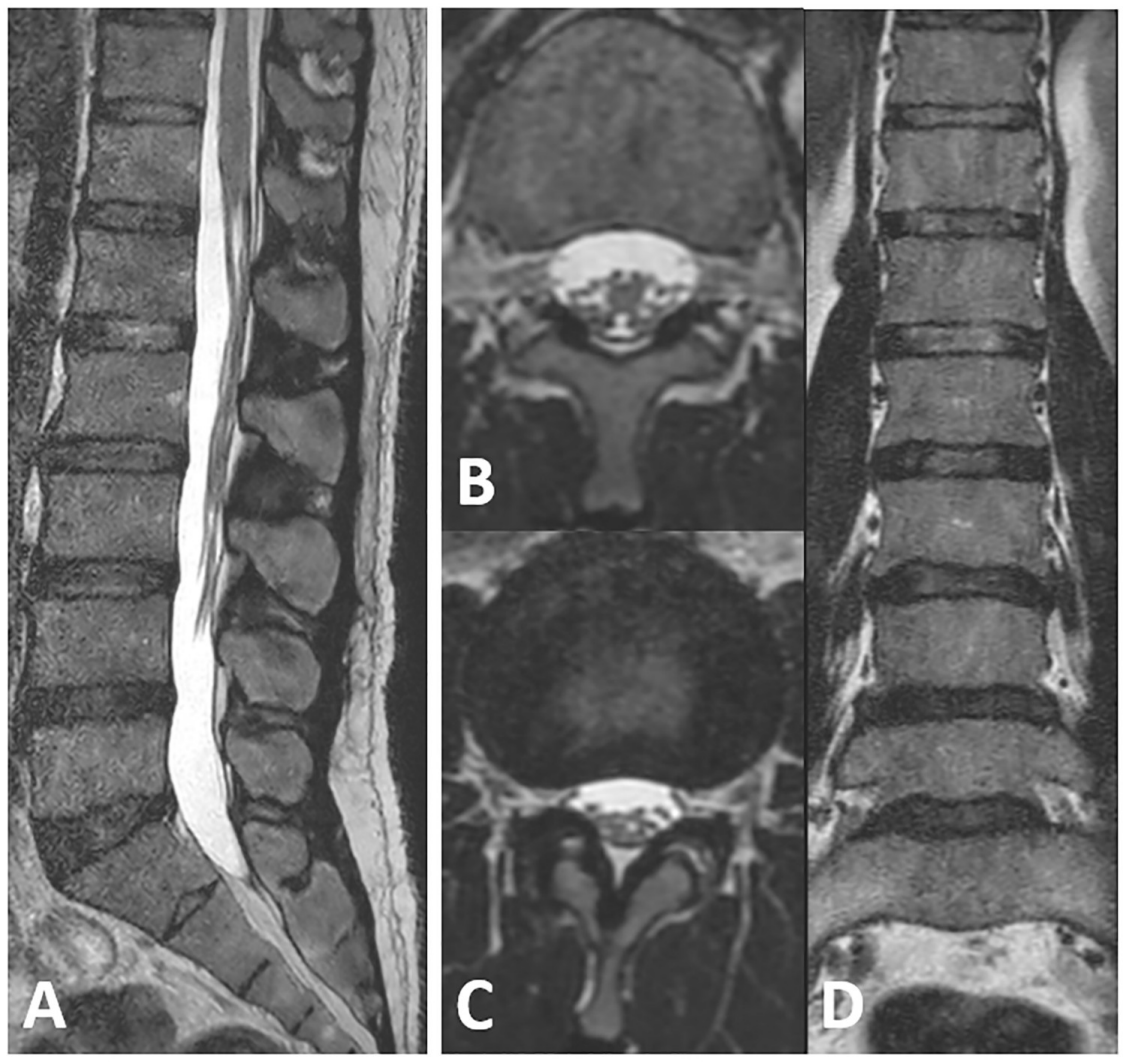
3D T2 spine view in lumbar spine. Sagittally scanned high resolution 3D T2 spine view of the lumbar spine acquired with nearly isotropic voxels and Compressed SENSE in a patient with spinal canal stenosis (A). Secondary multiplanar transverse (B and C) and coronal reconstructions (D) were performed.

In the knee, lumbar spine and wrist protocols a hybrid approach was taken, where some scans were accelerated, and some 2D scans acquired in multiple orientations were replaced by a 3D scan of isotropic or near-isotropic resolution in combination with multi-planar reformats of this scan in other orientations (Fig 5 for example images of the lumbar spine).

The time reduction of individual scans ranged from 23–43% across 2D and 3D scans (32.3 ± 5.8%), with lower acceleration capabilities in 2D scans (range 23–35%; 29.6 ± 4.1%) compared to 3D scans (range 32–43%; 37.8 ± 5.1%) (p = 0.013).

The gains in the theoretical total exam time range from 13% in breast and wrist to 38% in knee joints. Over the selected exams, an average acceleration of 26.0 ± 11.2% was achieved, without taking the procedure mix into account.

### Log file analysis

**Fig 6:**
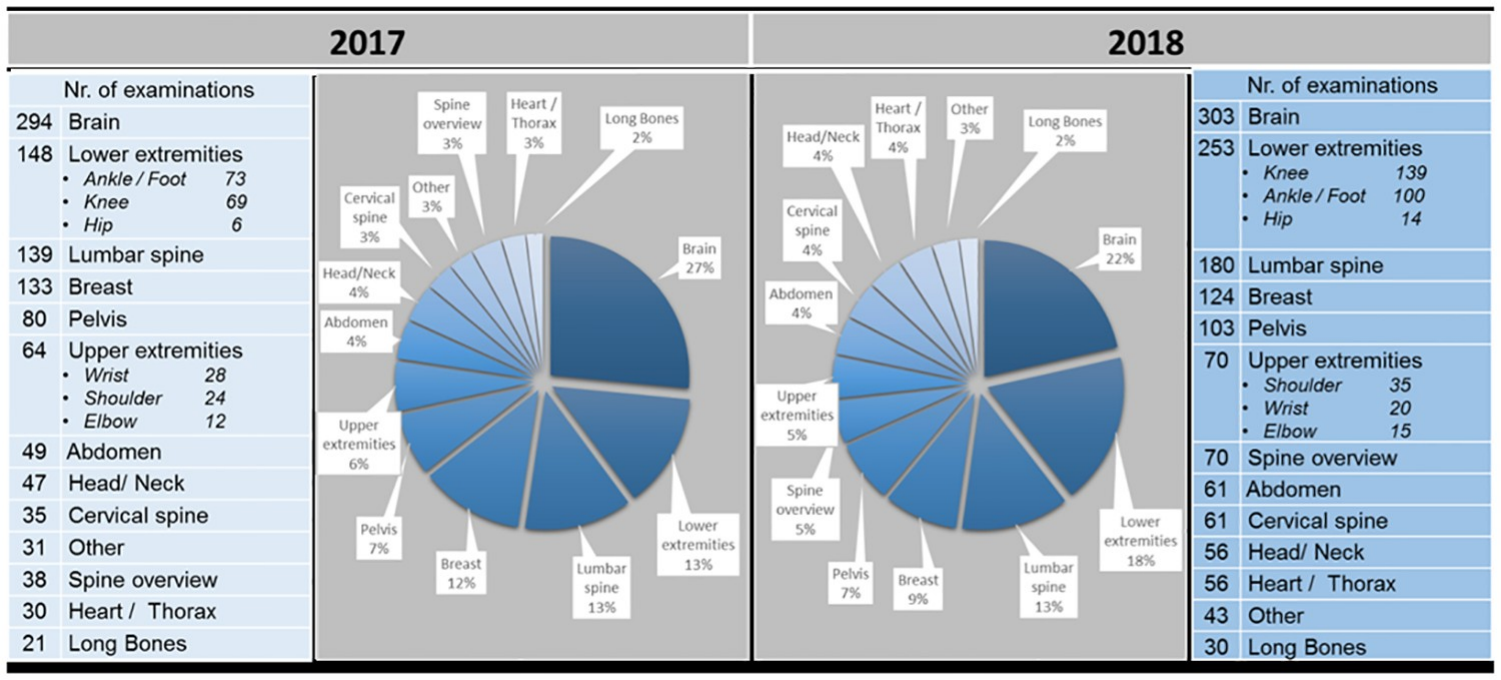
The mix of MR procedures. The mix of MR procedures before implementation of Compressed SENSE from January to April 2017 as well as after implementation of Compressed SENSE from January to April 2018. All MR examinations based on standard and non-standard protocols in all anatomical regions were included.

In 2018, the mix of all MR procedures related to anatomical regions was similar compared to 2017 as depicted in Fig 6.

Especially the most important MR examinations concerning brain, lower extremities (including knee), lumbar spine, breast and upper extremities (including wrist and shoulder) had a comparable frequency.

Considering only the six selected anatomical regions, in 2017, 83.9% of the MR protocols used were standard protocols. In 2018, after the implementation of Compressed SENSE, standard protocols represented 90.6% of the MR protocols used for the six regions.

**Fig 7:**
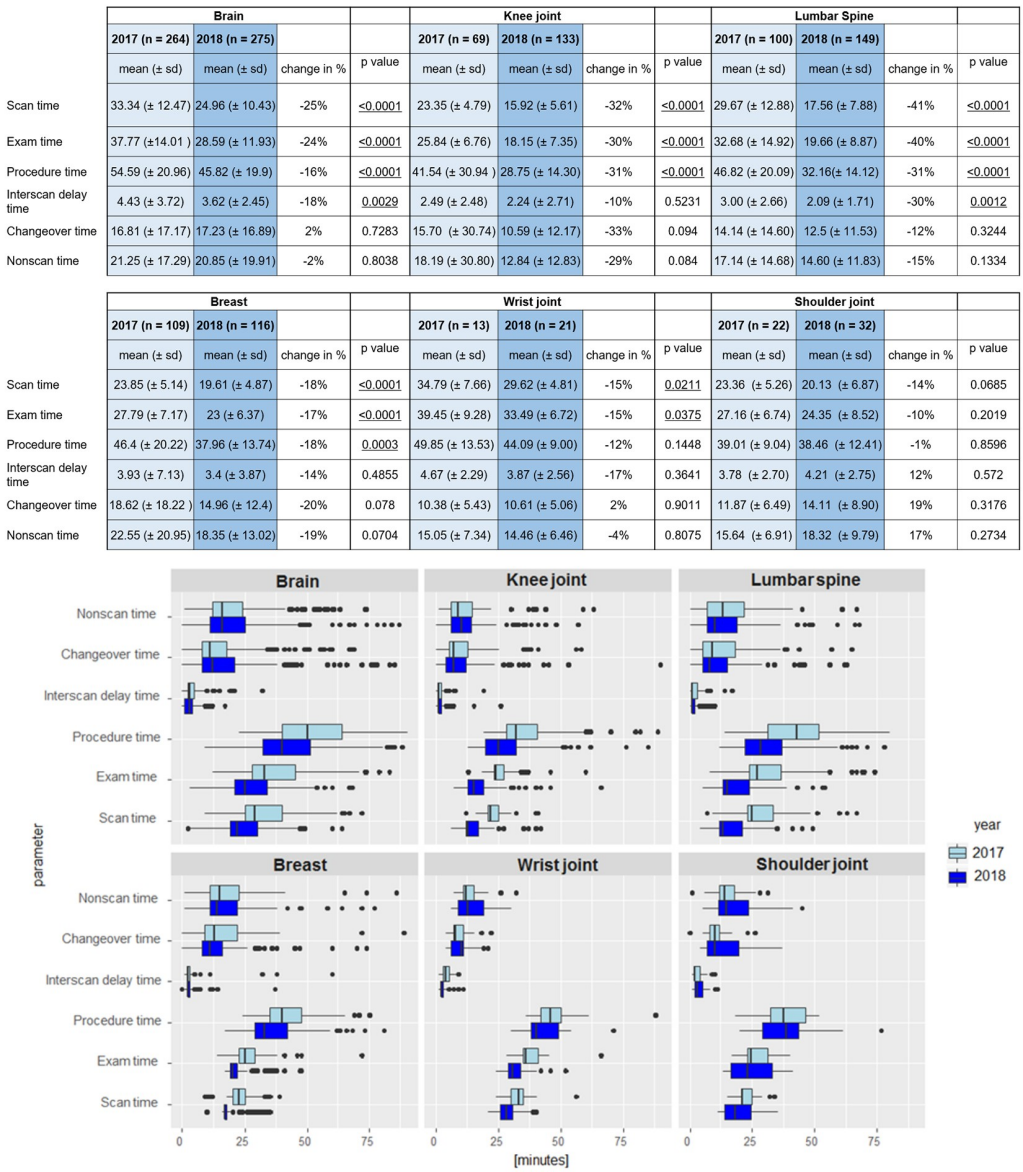
Exam timing parameters derived from the MR log files. All exam timing parameters are depicted in minutes for the 6 selected anatomical regions (brain, knee, lumbar spine, breast, wrist and shoulder (Fig. 7a). Only the standard protocol of each anatomical region was considered. The same data is depicted in boxplots (Fig 7b).

In the exams of 2018 where Compressed SENSE was applied (considering only standard protocols of the 6 anatomical regions as depicted in Fig 7a and 7b), reductions in scan time of 14% (shoulder) to 41% (lumbar spine) were achieved with averages of 24.2% ± 10.7%.

The exam times were affected with similar percentages: -10% (shoulder) and -40% (lumbar spine) with averages of -22.7% ± 11.0%. More importantly, the procedure times that mark the duration of the patient being in the exam room, were shortened by 1% (shoulder) to 31% (knee and lumbar spine) with averages of 18.2% ± 11.5%. The reduction in scan and exam times were significant in all anatomical regions except for the shoulder. Procedure times were significantly shortened in all regions except wrist and shoulder. Next to reductions in scan, exam and procedure times, changes are observed in nonscan time. In all regions except shoulder the nonscan time is reduced, but in none of the anatomies this yields a significant effect. Also in the largest component of nonscan time, the changeover time, no significant changes were observed. Only in the minor component of nonscan time, the interscan delay times, significant reductions in time were observed in brain and lumbar spine.

**Fig 8:**
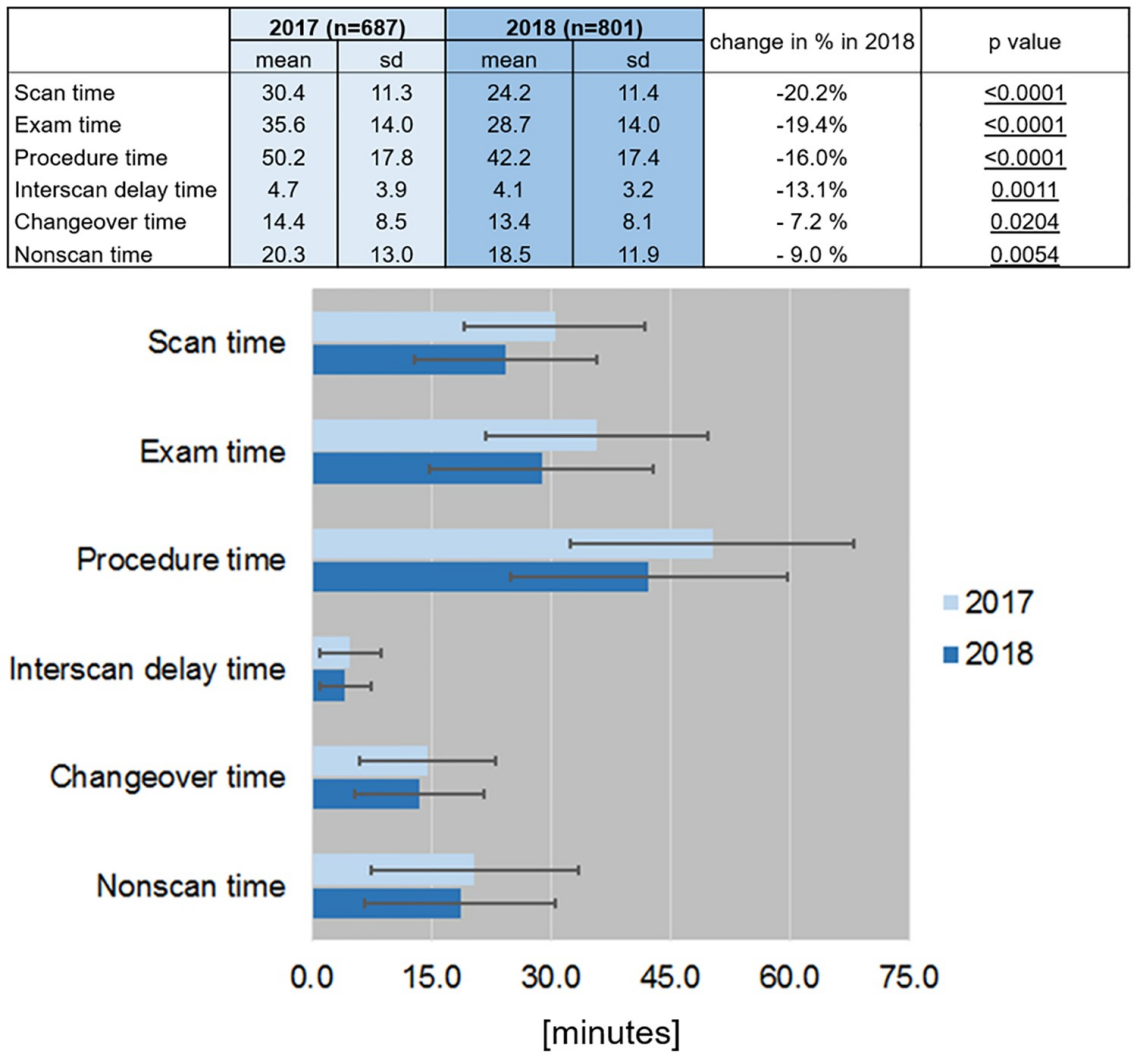
The average exam timing parameters. The average exam timing parameters for the 6 selected anatomical regions as derived from the log files, depicted in numbers and in bar plots. All MR examinations based on standard and non-standard protocols of the respective anatomical region were considered.

However, when all MR examinations in the time interval from January to April 2017 were compared to all MR examinations in the time interval from January to April 2018 a statistically significant time reduction in all examination timing parameters was present (Fig 8).

These reductions varied from 7.2% in changeover time to 20.2% in scan time. An overall gain in procedure time of 16.0% was achieved in the same 4 month period in 2018 compared to 2017.

**Fig 9:**
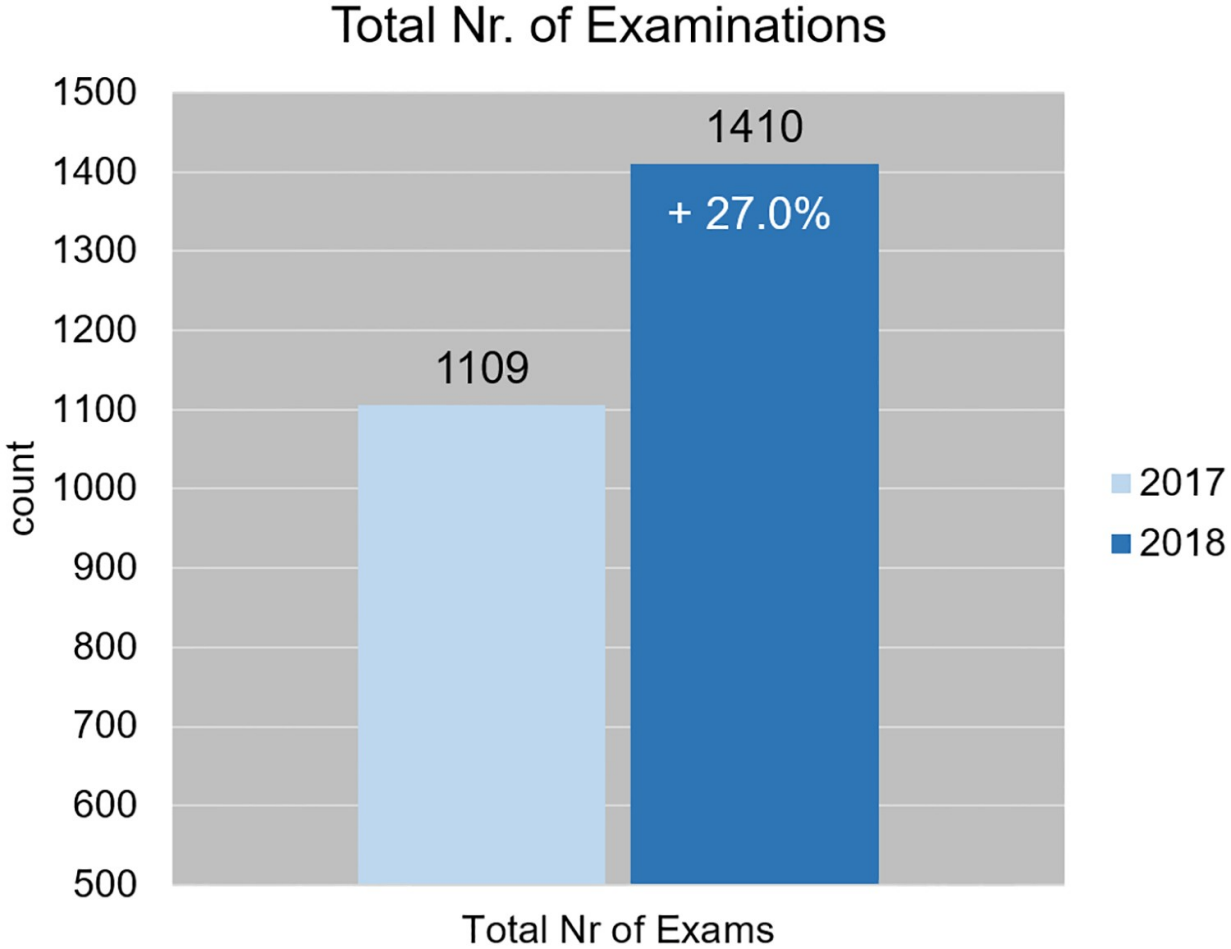
The total number of MR examinations. The total number of MR examinations obtained in the selected 4 month time period. All MR examinations based on standard and non-standard protocols in all anatomical regions were included.

Finally, the total number of MR examinations (considering all MR protocols and all anatomies) performed at our 1.5 Tesla MR scanner was increased by 27% if time intervals from January to April 2017 and 2018 were compared as demonstrated in Fig 9.

Based on a 12.5 hour workday and 85 working days in the period, this resulted for 2017 in an average of 13.0 patients per day in time slots that were on average 57.6 min. In 2018, a total number of 1410 patients were scanned in the same period, resulting in an average of 16.6 patients per day and average time slots of 45.2 min.

## Discussion

In this study, we investigated whether previously reported acceleration possibilities of Compressed SENSE [2] are achievable in the routine clinical radiological setting. Following installation of Compressed SENSE at our institution, imaging protocols were first redesigned either by reducing acquisition times with unchanged and uncompromised image impression or by improving spatial resolution moving from 2D acquisitions in multiple scan orientations to 3D approaches with (close to) isotropic resolution and multi-planar reformats with secondary reduction of acquisition times. In individual scans that were suitable for Compressed SENSE, an acquisition time reduction of individual sequences of 23–43% across 2D and 3D scans was achieved. In line with expectations, higher acceleration factors were achieved in 3D scans as there is more room for aggressive undersampling compared to in 2D scans. In comparison to previous results, this almost matches with the expected reduction of up to 50% for individual sequences. We did observe differences with the exemplary acceleration factors published by the vendor, in some anatomies we did not achieve the same numbers, such as in brain imaging, where we achieved 25% acceleration compared to 45% predicted. In other anatomies we observed much higher acceleration factors, for example in lumbar spine where 25% was predicted, but in our case 41% acceleration was realized supporting previously published data of a scan time reduction of 45% for a 3D T2 TSE sequence of the lumbar spine [10]. These differences can be explained by the fact that the total acceleration factor is highly dependent on the protocols in the a priori situation, and therefore it is difficult to predict total acceleration factors of exam protocols without knowledge of the local situation and starting protocols. Protocol optimization resulted in improved exam times for all six selected anatomical regions. The total acceleration times on average result in a speed-up of 26%, ranging from 13% in wrist to 38% in knee. It has to be stressed that these acceleration percentages are theoretical values that can be achieved based on the implementation of Compressed SENSE, without taking into account other factors that influence procedure times such as changeover times as well as the fact that next to these predefined protocols other indication based protocols and sequences are used in clinical practice. Furthermore, the procedural mix of the different MR protocols is not taken into account to calculate the acceleration percentages mentioned here.

The log file analysis resulted in concrete data whether the theoretic acceleration factors were achieved over a 4-month period in clinical practice. Scan times were expected to have similar amounts of accelerations as predicted from the designed exams, while exam times and procedure times were also influenced by other factors. These are related to the efficiency of the radiographers with respect to patient positioning (changeover time), patient movements (with the need to repeat scans) and planning and communication with the patient during the exam (interscan delay time).

For the standard protocols of the six anatomical regions, the changes in nonscan time were not significant. However, considering all protocols, a significant reduction of 9% was achieved probably due to the fact that radiographers had to perform patient handling more efficiently due to shorter imaging slot times.

In our institution, over the six selected anatomical areas, we achieved an average exam time reduction of 19.4% and an average reduction of 20.2% of scan times taking into account all examinations performed in the observed period for these anatomical areas. Shoulder imaging was a clear outlier, where a theoretic scan time reduction of 31% based on acquisition times in the exam card of the standard shoulder protocol was expected, but in practice, a scan time reduction of 14% and an exam time reduction of 10% of the standard shoulder protocol was achieved. The t-test for the exam and scan time did not reveal significance. This may however be caused by the small sample size of standard shoulder protocols. The lower time reductions (as also observed for knee joint) are explained by the fact that sequences had to be repeated quite often due to patient movements caused by severe pain.

Generally speaking, there were slight differences for the standard MR protocols between the theoretical acceleration factors as calculated from the exam cards and the real acceleration factors in the clinical setting. Sometimes our data even showed that the real acceleration factor was greater than predicted, as in the case of lumbar spine (37% vs 41% acceleration). This may be explained by the fact that examinations were terminated mid-way due to patient inconvenience or that not the whole standard protocol was completed for reliable diagnosis.

Ultimately, the procedure time is the most important factor for the management of the radiology department, as this determines the duration of the time slots and the amount of patients that can be scanned per day. We did observe an average decrease in procedure times of 16.0% in all MR examinations of the six anatomical regions in the observed period. Again, this number is slightly skewed by the bad performance of the standard protocol in shoulder imaging (-1%), while in other anatomies larger reductions in procedure time were observed, such as 31% improvement in both knee and lumbar spine for the standard protocols.

Given these results, the implementation of Compressed SENSE allowed us to reduce imaging slot times. The Brain MRI time slot went from 45 min to 35 min, knee MRI from 30 min to 20 min, lumbar spine MRI from 30 min to 20 min, breast MRI from 45 min to 30 min and wrist MRI from 45min to 35min. Time slots for shoulder imaging were maintained at 30 min. Additionally slot time went down from 30 min to 20 min in cervical spine, from 60 min to 45 min in abdomen and from 60 min to 45 min in head and neck exams.

When only examining all MR examinations performed for the six anatomical regions that have been analysed in detail, 16.6% more examinations were performed in 2018, which corresponds well with the 16% acceleration of procedure times in these selected MR examinations. Due to significant accelerations of scan times in additional anatomical areas through application of Compressed SENSE, as for example in cervical spine, in brachial plexus, in abdomen and head and neck exams a final increase of 27% in total MR examinations in the selected period in 2018 was achieved compared to the same period in 2017. This translates to 3.6 more patients per day on average.

We have not yet fully exploited the potential of Compressed SENSE in all sequences. Some sequences have not yet been accelerated, although technically possible. The reason is simply a lack of time for the adaptation of the protocols in a busy daily clinical schedule. Limitations of the study are that our results are not fully reproducible at another institution as the initial MR protocols and the reduction factors and denoising levels chosen show variations. Furthermore, other institutions may choose to optimize the MR protocols in a different manner. Moreover certain examination timing parameters (i.e. changeover time) depend on the efficiency and experience of the radiographers and this factor was not specifically controlled or corrected for between the selected periods. Analysis was limited to six body regions and to examinations performed on 1.5T MR scanner. Apart from our initial image quality assessments described in this study, other studies confirm the unchanged image quality if sequences are scanned with Compressed Sensing techniques [11–14]. Especially in a neuroradiological and musculoskeletal setting the image impression and quality was described as comparable in comparison to images acquired without Compressed SENSE if denoising level and Compressed Sense factor were chosen accordingly [11–14].

Possible artefacts as reported by Sartoretti et al. [11] may occasionally be encountered in some sequences, yet do not impair the diagnostic reliability.

The implementation of Compressed SENSE has allowed us to significantly shorten all MR examination timing parameters for the six anatomical regions analysed, resulting in shorter time slots for 5 out of 6 anatomical regions and therewith enabling an increase of 16.6% in examinations in a 4-month period in 2018 compared to 2017. The overall increase of patient volume was 27% as Compressed SENSE was applied in other anatomies as well, that were not taken on in this detailed assessment.

In conclusion, the implementation of Compressed SENSE has allowed us to significantly shorten all examination timing parameters for the six anatomical regions analysed, resulting in shorter time slots for 5 out of 6 anatomical regions and therewith enabling an increase of 16.6% in examinations in a 4-month period in 2018 compared to 2017. The overall increase of patient volume was 27% as Compressed SENSE was applied in other anatomies as well, that were not taken on in this detailed assessment. A substantial optimization of the clinical workflow was achieved without the help of additional staff and thus without additional costs. Therefore the net revenue of the institute could be increased considerably. Ultimately patients greatly appreciated the shorter examination times while also benefiting from being examined after a much shorter waiting period.

## Supporting information

S1 File Data supporting the manuscript. (XLSX)

S2 File Data supporting the manuscript. (XLSX)

S3 File Data supporting the manuscript. (XLSX)

## Abbreviations

CT: computed tomography
DIR: double inversion recovery
DRIVE: driven equilibrium
DWI: diffusion weighted imaging
FFE: fast field echo
FLAIR: fluid attenuated inversion recovery
IQR: interquartile range
N.A.: not available
PC: phase contrast
sqrt: square root
SD: standard deviation
SENSE: sensitivity encoding
STIR: short tau inversion recovery
SPAIR: spectral attenuated inversion recovery
SPIR: spectral presaturation with inversion recovery
T1w: T1 weighted
T2w: T2 weighted
TE: echo time
TFE: turbo field echo
TOF: time of flight
TR: repetition time
TSE: turbo spin echo
